# Direct patterning of organic conductors on knitted textiles for long-term electrocardiography

**DOI:** 10.1038/srep15003

**Published:** 2015-10-08

**Authors:** Seiichi Takamatsu, Thomas Lonjaret, Dakota Crisp, Jean-Michel Badier, George G. Malliaras, Esma Ismailova

**Affiliations:** 1 National Institute of Advanced Industrial Science and Technology, Tsukuba 305-8564, Japan; 2 Department of Bioelectronics, Ecole Nationale Supérieure des Mines, CMP-EMSE, MOC, 13541 Gardanne, France; 3 MicroVitae Technologies, 13590 Meyreuil, France; 4 Aix Marseille Université, INS/Inserm, UMR-S 1106, 13005 Marseille, France

## Abstract

Wearable sensors are receiving a great deal of attention as they offer the potential to become a key technological tool for healthcare. In order for this potential to come to fruition, new electroactive materials endowing high performance need to be integrated with textiles. Here we present a simple and reliable technique that allows the patterning of conducting polymers on textiles. Electrodes fabricated using this technique showed a low impedance contact with human skin, were able to record high quality electrocardiograms at rest, and determine heart rate even when the wearer was in motion. This work paves the way towards imperceptible electrophysiology sensors for human health monitoring.

Textile-based health monitoring devices are receiving a great deal of interest for consumer and medical applications^1^,^2^,^3^,^4^,^5^,^6^, where they are being used to monitor parameters such as blood pressure^7^ and heart rhythm^5^,^6^. The main advantage of textiles as substrates for biomedical devices stems from the fact that they establish and maintain conformal contact with the human body in a non-invasive way^1^,^2^,^3^. Indeed, shirts^1^,^4^, gloves^8^, and wristbands^3^ outfitted with sensors have been used to demonstrate the potential of this technology. There is an increased interest in the development of electrocardiography (ECG) electrodes, to which textiles can grant the conformal skin contact which is necessary in order to accurately detect the small electrophysiological signals of the heart^4^,^5^,^9^,^10^. Wearable ECG electrodes can enable the remote monitoring of people at risk, signal the onset of heart disease, and help monitor physical activity during exercise^11^. Despite the large interest in cutaneous electrodes, patterning conducting materials on stretchable fabrics has been hampered by their three-dimensional nature, which makes difficult to apply conventional patterning processes^1^,^12^,^13^,^14^. The choice of the pattern transfer technique is defined by the textile’s type and its structure. The yarns in woven and non-woven fabrics are interlaced in a dense net, resulting in very flat, but mostly non-stretchable, structures^15^. The fibers in knitted textiles are assembled in a snake shape that can be altered by applying a mechanical force to the knit varying its design. Such wave-form fibers mimic a mechanical spring design, providing the textile with a considerable resisting force when its shape is changing. Usually direct pattern transfer can be done on thin woven and non-woven textiles, while embroidery and knitting are used to pattern thick and structured textiles.

Micro-contact, inkjet, and screen printing can be used for making conductive patterns on textiles^16^,^12^. Therefore, the controlled transfer of a pattern using these techniques on thick knitted fabrics can be obstructed. The conductive materials need to be coated not only on the surface of the knitted structure but also inside, providing continuous contact between the yarns during its mechanical deformation. Micro-contact and inkjet printing allow direct pattern transfer which is usually done on thin textiles since a small amount of the ink can be transferred at the time. The coating happens only on the top layer of the textile however the pattern conductivity is maintained even under stretch. In the screen printing, additives are used to reduce the spreading of the ink (i.e., silver paste) and improve spatial resolution^17^,^18^. The viscosity of inkjet printing inks must also be engineered for printability^19^. As a result, in both screen- and inkjet printing, ink optimization has a negative impact on the final conductivity.

Embroidery and knitting consist of using individual fibers and subsequently introducing them into the textile’s structure^14^,^20^,^21^,^22^. Thin stainless steel, copper or other metal wires are employed to sew conductive patterns on textiles by embroidery. A large amount of wires is needed during these processes to create a pattern. Usually such techniques are largely integrated in textile industries to create interconnections between sensors and output electronic systems. Additionally, organic electronic materials can be also applied via die-coating, where the fiber gets coated by traveling through a nozzle filled with a conducting material and then weaved or knitted during textile manufacturing. Examples of this approach include large area touch sensors^14^, electric wiring components^13^, and organic electrochemical transistors^15^ using organic conducting material such as poly(3,4-ethylenedioxythiophene):poly(styrene sulfonate) (PEDOT:PSS). Electrodes made of PEDOT:PSS have been successfully used in cutaneous applications highlighting their high performance compared to commercial ones. With proven biocompatibility, the material has been used in *in vivo* studies and was shown to decrease the electrical impedance over classical electrodes^23^. Most importantly, this commercially available polymer can be easily modified without compromising its practicality. PEDOT’s rheological properties make it attractive for direct integration with textiles. A key challenge, therefore, for the elaboration of biomedical devices on textiles is the development of simple patterning techniques that allow the deposition of conducting biocompatible materials only on a desired area, with neither the need for cutting and sewing nor for additives that affect conductivity.

In this work, we report a simple and reliable fabrication process that allows the patterning of conducting polymers on thick knitted textiles, thereby yielding wearable and conformal electronic devices for healthcare monitoring. We applied this process to the fabrication of cutaneous electrodes using the commercially available, high conductivity polymer PEDOT:PSS, and an ionic liquid gel that promotes better skin contact. We evaluated the performance of these devices in electrophysiological recordings of a human heart. The measurements showed that the textile electrodes form a low impedance contact with skin and are able to capture the electrophysiological signal with high accuracy, even during motion. These results pave the way for the simple fabrication of a variety of biomedical devices on textiles.

## Results

### Patterning technique

**Figure 1:**
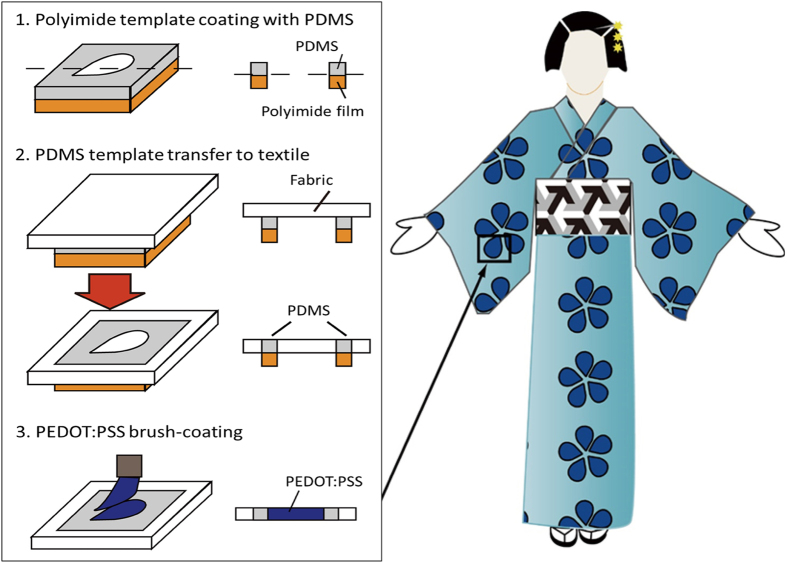
Process flow for the patterning of PEDOT:PSS on textiles, inspired by the Japanese kimono dying method. PDMS is first deposited on a polyimide master defining the outline of the desired pattern. The textile is then placed on the polyimide film and the PDMS is progressively transferred into the bulk of the textile. After a short thermal annealing, the PEDOT:PSS solution is brush-coated on the unprotected area of the textile and dried.

**Figure 2:**
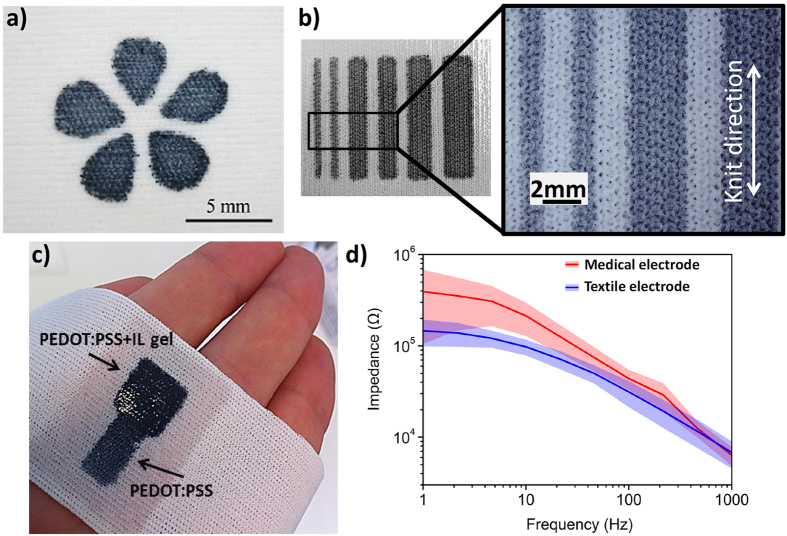
Patterning results. (**a**) Flower pattern of PEDOT:PSS on a textile (in dark blue) and (**b**) a “lines and spaces” test pattern with lines of 1, 2.5, 3 and 4 mm width, with a magnified view of the 1 and 2.5 mm lines. (**c**) Photograph of a textile wristband with a PEDOT:PSS electrode. The electrode has an area of 1 cm^2^ which is also coated with an ionic liquid (IL) gel, indicated by PEDOT/PSS +IL gel arrow. A connection pad is also visible and indicated by PEDOT:PSS arrow. (**d**) Impedance spectra of a textile and a medical electrode of similar area measured in the 1 Hz to 1 kHz frequency range in contact with skin from three different volunteers (mean (straight line) and standard deviation (confidence area)).

The technique was inspired from the Japanese kimono dyeing process (the Yuzen method). According to this method, a rice paste is initially painted on the textile surface to form a stencil. The dye is subsequently applied, coating only the areas that are free of rice paste which is subsequently removed in water, resulting in the precise and beautiful patterns of a kimono. In Fig. 1 we show the adaptation of this technique to the patterning of PEDOT:PSS on textiles. We used polydimethylsiloxane (PDMS) as the stencil due to its hydrophobic nature, which can confine the aqueous PEDOT:PSS solution, as well as due to its mechanical properties, which are a good match for the soft and stretchable structure of knitted textiles. The patterning process started by preparing a negative master made of a polyimide (PI) film on which the outline of the desired pattern was carved by a laser cutting machine. PDMS was subsequently applied on this master by spin coating (step 1). The textile was then placed on top of the PI master, and the PDMS was progressively transferred into the textile (step 2). By adjusting PDMS viscosity (using different amount of the curing agent) and thickness (using different spin coating speeds) it is possible to control its diffusion into the textile and to replicate the master design. A short thermal annealing was used to cure the PDMS completing the transfer step. The PI master was then delaminated from the textile surface. Finally, the conducting polymer solution was brush-painted on the textile and baked to dry (step 3). Contrary to the Yuzen method, in which the rice paste is removed, the PDMS stencil remains in the textile after the patterning process, and can be used to pattern additional layers (see below). Typical patterning results can be seen in Fig. 2. The textile used in this study was a knitted polyester with a thickness of 300 μm and a stretch capability up to 50% (in the knit direction). A flower pattern with regularly spaced curved features and a lines-and-spaces test pattern are shown in Fig. 2a,b, respectively. From the latter it can be seen that the resolution is greater than 1 mm, which is adequate for cutaneous electrodes. Additionally, the patterning on different textiles is shown in Suppl. Fig. S1. These results show that patterning of structures as fine as 0.5 mm is possible on tightly woven textiles. The sheet resistance of the patterned PEDOT:PSS stripes was 230 Ω/sq. This value was the same as that of a dip-coated textile, showing that there is no influence of the patterning process on the electrical properties of the conducting polymer.

### Electrode fabrication

Following the described fabrication process, 1 cm^2^ PEDOT:PSS electrodes were patterned on knitted polyester. Fig. 2c shows the final structure of an electrode integrated on the polyester wristband. The deposition of the PEDOT:PSS can be followed by the deposition of additional layers, which will also be patterned using the PDMS stencil. In this particular case we added an ionic liquid (IL) gel, as these materials are known to help establish low-impedance cutaneous contacts^24^. By adding about 45 μL/cm^2^ of IL gel formulation, patterning was achieved on just the active area of the electrode. The wristband containing the PEDOT:PSS/IL gel electrode was wrapped around the arm of a volunteer and skin impedance was measured and compared to that of a hydrogel-assisted Ag/AgCl medical electrode with a similar active area. The measurements were carried out on three different volunteers in rapid succession, on the same spot. The results, shown in Fig. 2d, follow the typical trend of the electrical impedance measured on skin^24^, and take into account subject to subject skin condition variations, represented by confidence area of the mean impedance curve. They reveal a lower impedance for the textile electrode (2.5 times lower at 10 Hz), paving the way for applications in cutaneous electrophysiology.

### Evaluation of electrodes in ECG monitoring

**Figure 3:**
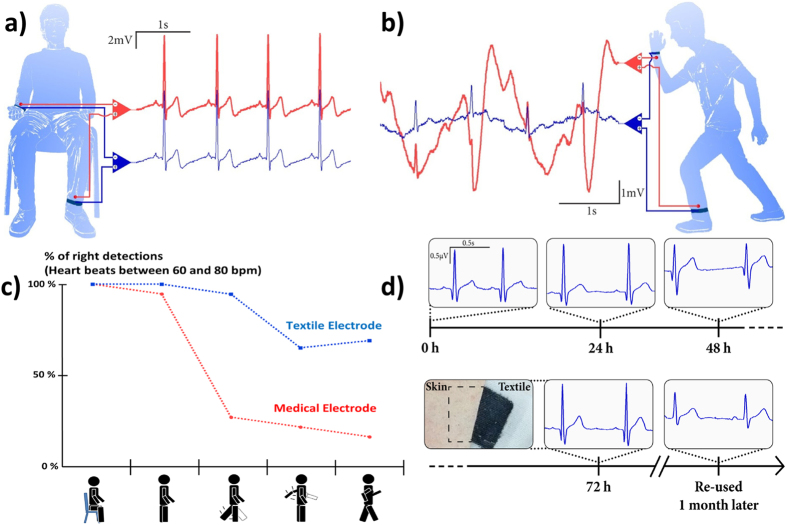
Electrodes evaluation in electrocardiography. ECG recordings performed with the PEDOT:PSS textile electrode (in blue), and a medical Ag/AgCl electrode (in red), (**a**) from volunteers sitting at rest, (**b**) during movement. (**c**) Percentage of accuracy of heartbeat detection during different types of activity (seating, standing up, leg moving, arm moving, walking) with medical and textile electrodes during a 50 s epoch. (**d**) ECG signal evolutions obtained with textile electrodes in permanent contact with skin over three days. The inset shows a picture of the skin under the electrode after 72 h. The last ECG signals were obtained from re-used textile electrodes stored in ambient air for one month.

The potential of the textile electrodes in biomedical monitoring was assessed using electrocardiography recordings. ECG is a common diagnostic technique in the clinic, and is also used to monitor heart rate during exercise. ECG measurements in dynamic conditions are usually performed with electrodes placed on the chest, in order to reduce motion artifacts. To evaluate the performance of the textiles electrodes in a wearable configuration, we used the limb lead II configuration (one electrode worn on the right wrist and a second one worn on the left ankle)^25^ and performed measurements on volunteers at rest and various states of movement consecutively during 3 hours. In this study, conventional medical electrodes were placed next to the textile ones for comparison. The results are presented in Fig. 3. During a first measurement, the volunteer was sitting at rest, to reduce muscle movements and respiratory artifacts. This position allows to obtain high quality recordings that can be used to detect heart function anomalies. Both, the textile and the medical electrodes show the typical waveform of the heart activity with similar amplitudes (Fig. 3a). High resolution PQRST complexes, corresponding to the different phases of polarization and depolarization of cardiac cells, can be clearly detected with both electrodes. To compare the signal quality recorded from textile electrodes with medical electrodes, SNR values were calculated after filtering in the 0.5 Hz–40 Hz frequency band (typically used in ambulatory patient monitoring). A feature recognition program first isolated each PQRST complexes and then found the Crest Factor, or peak-to-RMS ratio, which is the ratio between the amplitude of the R peak^26^ (which is the peak with the highest amplitude) and the RMS value of the signal all along the complex. The SNR value is averaged over 25 different complexes obtained with textile and medical electrodes and found to be equal to 16.3 dB (±0.1 dB) for both electrodes.

### Evaluation of electrodes under ambulatory conditions

The evaluations were performed first in the movement and then during long-term experiments. Fig. 3b shows recorded signals obtained while the volunteer was standing up and moving. Movement can have a large impact on ECG recordings^27^, inducing an undulating baseline that can disturb the acquisition of the PQRST complex. This is evident in the recording obtained by the medical electrode (in red), where the R peak is barely visible. In contrast, the influence of motion is significantly lower on the recordings from the textile electrode (in blue), which show richer signal content, with a well-defined R peak and even a visible T wave (the positive wave following the R peak). We calculated that the baseline noise (low frequencies) is 13.1 dB higher for the medical electrode. As a result, a standard algorithm for the calculation of heartbeat frequency fares considerably better with recordings from the textile electrode. On a subject with a mean heartbeat of 70 bpm (at rest or during low-level activity), Fig. 3c demonstrates that R peaks are more accurately detected in recordings obtained with the textiles electrodes for a panel of different types of motion.

The long-term signal stability is assessed by continuously placing two textile electrodes on a volunteer’s chest during 3 days. ECG recordings from these electrodes are presented in Fig. 3d and demonstrate that the signals are highly consistent during this recording time. The SNR and R-Peak amplitude evolutions are presented in Table S1. Despite the signal variations from day to day related with the skin hydration changes and environmental noise, their amplitude and noise level remain stable. There was no skin reaction to the electrodes observed after 3 days. Moreover, the same electrodes were stored in ambient air for 1 month and then were re-used in ECG recordings in the same setup. These electrodes were still able to record well defined PQRST complexes, highlighting the long-term stability of the textile PEDOT:PSS/IL gel electrodes.

## Discussion

The technique discussed here allows the patterning of conducting materials with a demonstrated resolution of 0.5 mm, a value that is adequate for the majority of envisioned biomedical applications. These include applications requiring small cutaneous electrodes, such as neonatal care and high-density electroencephalography, where the feature size is larger than several millimeters. In the described patterning process, we were able to combine the direct patterning with relatively thick knitted textiles. We have benefitted from the scalability of this approach and we adopted it for thick structured textiles in a time and cost efficient way. This technique makes use of industrially accepted techniques such as contact printing and deep painting, and can be easily applied not only during textiles manufacturing but also in post manufacturing by processing existing garments. PDMS and PEDOT:PSS materials that are used in the patterning are fully compatible with knitted textile platform thanks to their rheological properties. The rubber-like PDMS stencil integrated with textile conserves the mechanical freedom of its structure. The viscoelastic properties of PEDOT:PSS formulation allows to achieve homogeneous coating of elastic and flexible knitted textiles, in our case, polyester.

The combination of a low contact impedance provided by the PEDOT:PSS/IL gel electrode and of a conformable support provided by the textile is shown here to diminish the impact of motion artifacts, which paves the way for a variety of applications, including electromyography (EMG). EMG, which monitors the electrical activity of skeletal muscles, requires electrodes that can record signals during motion. The high tolerance of textile electrodes to low frequency motion artifacts makes them well-suited for this application. Finally, electroencephalography (EEG) is another obvious application for these textile electrodes. EEG measurements are currently performed using electrodes mounted on casks, then filled with gel. The monolithic fabrication of a cask with integrated electrodes will make these measurements easier to perform. Such measurements are currently ongoing in our own lab. As textile electrodes can be easily integrated with hats, they can be used to render EEG electrodes imperceptible to the wearer. As a result, this can increase the acceptance of EEG in applications beyond healthcare, such as gaming and fatigue monitoring.

Existing commercial electrodes are assisted with hydrogels recognized as biocompatible systems. However, some people manifest cutaneous reactions to such gels after long contact. It’s very important to underline that the skin condition as well as the skin sensitivity dramatically vary from subject to subject. Chemical engineers are constantly pursuing the development of novel and more tolerant chemicals that can be used for cutaneous electrophysiology. Ionic liquid gels are fully cross-linked systems providing solid electrolytic contact with the skin required in cutaneous electrophysiology. Generally the toxicity of ionically charged systems originates mainly from the cation. Novel formulations of ionic liquids with large and bulky cations that are entrapped in polymeric matrix propose stable and biocompatible gel systems. The process used in this paper can serve as a model for integrating such systems with conductive textiles. The toxicity of this material in not fully demonstrated yet. In our experiments we have not observed any skin irritations during electrophysiological experiments performed over a 3 day period.

The technique developed here is generic and would work with any material soluble in aqueous media, provided that post-processing (annealing, sintering, etc.) temperatures stay within the range that the textile can support. The ability to pattern a second layer on top of the conducting polymer paves the way for the development of a wide variety of devices, including organic electrochemical transistors that can be used for simple logic circuits^28^, endowing textiles with signal processing capabilities. It also includes biosensors, which, in the simplest configuration, consist of a conducting polymer and a redox enzyme-containing gel. Such biosensors can be used for the detection of metabolites including glucose and lactate in sweat^29^, thereby providing information about blood sugar level and muscle fatigue. In addition to applications in healthcare (along the lines of a “smart” bandage), such sensors will extend the scope of bioelectronic textiles to areas including sports and recreation. Finally, the integration of energy and communication modules on textiles represents an important step in the evolution of this technology. Components such as batteries and antennas, which can be made out of conducting polymers^30^, can be patterned in a straightforward way with the technique developed here.

In conclusion, we developed a technique that allows the simple patterning of conducting polymers on knitted textiles. The technique uses a PDMS stencil to confine the spreading of the polymer to dimensions as small as 0.5 mm. PEDOT:PSS electrodes fabricated this way and coated with an ionic liquid gel showed a low impedance contact with the skin. They were able to record high quality electrocardiograms in clinic and ambulatory conditions, and accurately determine heart rate, even when the wearer was in motion. Moreover, these electrodes demonstrated high performance long-term stability during 3 days of ECG recordings and after extended ambient storage without any special reconditioning.

## Methods

### Patterning

The textile used was 100% interlock knit polyester fabric from VWR International (Spec-Wipe® 7 Wipers). The Kapton (HN100) polyimide film with 125 μm thickness was provided by DuPont. Laser cutting was achieved with a Protolaser S (LPKF) to pattern the polyimide mask. The PDMS formulation (RTV615, elastomer and curing agent kit) was purchased from Momentive Performance Materials and spin-coated on top of the polyimide at 550 rpm for 18 sec. The fabric was then gently transferred to the mask coated with PDMS. After 10 minutes, PDMS was fully absorbed in the textile structure which was then cured at 100 °C during 10 minutes for the first annealing step. The polyimide mask was removed before the deposition of the PEDOT:PSS solution. The second step of curing was applied to the fabric at 110 °C during 1 hour. The conducting polymer formulation consisted of 80 mL of PEDOT:PSS dispersion (CleviosTM PH1000, Heraeus), 20 mL of ethylene glycol (Sigma Aldrich), 40 μL of 4-dodecylbenzenesulfonic acid (Sigma Aldrich), and 1 mL of 3-methacryloxypropyltrimethoxysilane (Sigma Aldrich). The ionic liquid gel consisted of a mixture of the ionic liquid 1-ethyl-3-methylimidazolium-ethyl sulfate (Sigma-Aldrich), poly(ethylene glycol)diacrylate and the photoinitiator 2-hydroxy-2-methylpropiophenone at a ratio of 0.6/0.35/0.05, respectively. For the electrode of Fig. 2b we pre-coated the textile with 20 μl/cm^2^ of ionic liquid, then added 25 μL/cm^2^ of ionic liquid gel formulation and exposed to UV light (a UVGL-58 handheld UV Lamp set to 365 nm) to initiate crosslinking.

### Electrical characterization and ECG recordings

All volunteers provided informed signed consent to participate in the study. The sheet resistance of PEDOT:PSS on the textile was measured with a four-probe set-up: the coated fabric was placed on top of four equidistant copper electrodes, a constant current source was applied between the two outer probes. The voltage drop between two inner probes was recorded to calculate sheet resistance. Impedance was measured in a 3 electrode configuration (working and counter electrodes were placed two cm away from each other on the forearm, and the reference electrode was placed 30 cm away on the arm) using Sensor N medical grade Ag/AgCl electrodes (Ambu) with a 0.95 cm diameter gel-assisted area as reference and counter electrodes, as previously described by our group^24^. The textile electrode was compared to the Sensor N electrode, placed 2 cm apart. Spectra were acquired using an Autolab potentiostat, equipped with FRA module (Metrohm B.V.), applying sinusoidal voltage of 0.01 V. For the ECG recordings, we used TE/K50430-001 medical grade Ag/AgCl disk electrodes (Technomed Europe) with a 2 cm diameter. Textile and medical electrodes were placed on the wrist and the ankle of a volunteer and connected to a SandsResearch system using EA68 or EA136 amplifiers during the 3 hours of evaluation sessions. Signals were processed and filtered using LabVIEW (National Instruments) software with a third-order Butterworth filter (passband with low and high cutoff of 0.5 Hz and 40 Hz, respectively). To extract baseline from ECG signals we used a wavelet approach corresponding to a low-pass filter with a cutoff of 1.93 Hz). The results in Fig. 3c are based on the signals presented in Fig. 3a,b and three other motion conditions processed in the same way. The algorithm for the calculation of heartbeat (R peak) frequency is a feature extractor LabVIEW software (available in BioMedical Toolkit from National Instrument) with intern filters between 10 and 25 Hz. ECG signals in Fig. 3d were recorded with a portable and wireless RF-ECG2 acquisition system (from GM3 Corporation, intern passband with low and high cutoff of 0.16 and 100 Hz, respectively). The electrodes were in continuous contact with the skin during 3 days. ECG data were collected for 30 seconds every 3 hours during 3 days.

## Additional Information

**How to cite this article**: Takamatsu, S. *et al.* Direct patterning of organic conductors on knitted textiles for long-term electrocardiography. *Sci. Rep.* **5**, 15003; doi: 10.1038/srep15003 (2015).

## Supplementary Material

Supplementary Information
