# Supplementary material for: Direct patterning of organic conductors on knitted textiles for long-term electrocardiography
Source: Sci Rep. 2015 Oct 8;5:15003. doi: 10.1038/srep15003 (PMC4597223; doi:10.1038/srep15003)
Supplement: Supplementary Information [file srep15003-s1.pdf]

Supplementary Information

Direct patterning of organic conductors on knitted textiles for long-term electrocardiography

*Seiichi Takamatsu†, Thomas Lonjaret†, Dakota Crisp, Jean-Michel Badier, George G. Malliaras, and Esma Ismailova**

Supplementary Fig. S1. **Evaluation of the patterning resolution on two different type of textiles**. **(a)** Conventional lines-and-spaces patterns ranging from 1 mm to 6 mm and stripes of different widths on a Nylon woven ribbon; **(b)** Patterning of stripes of different widths on a knitted polyester fabric. The inset shows the width of a stripe being slightly larger than a single loop of the knitted fabric, which is around 500µm.

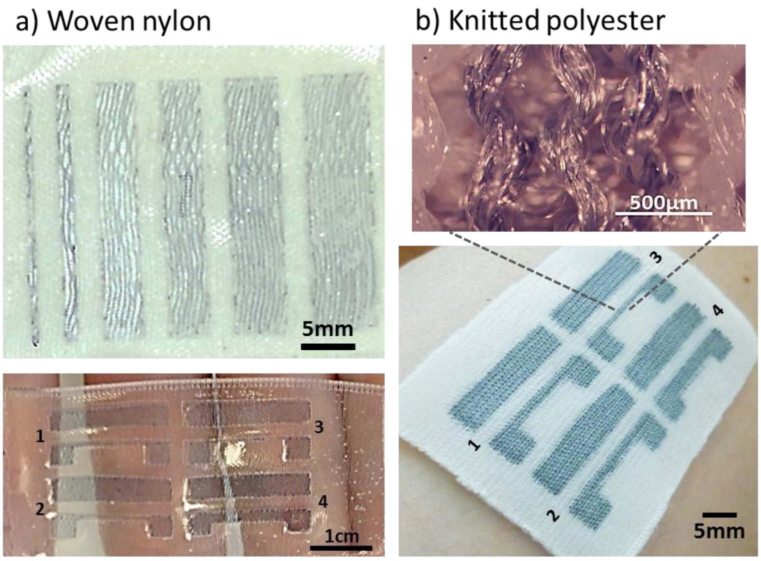

Supplementary Table S1. **Long-term evaluation of ECG signals obtained from textile electrodes**. R-Peak amplitude and Signal-to-Noise Ratio (SNR) were calculated from 30s epoch of ECG signal regularly collected during 3 days (mean (±standard deviation)). The last ECG signal was obtained from a re-used textile electrode stored on open-air for one month.

ECG SIGNAL EVOLUTION	Continuously in contact with the skin						Re-used
	0h	1h	12h	24h	48h	72h	1 month
R-Peak Amplitude (mV)	1.27 (±0.09)	1.13 (±0.05)	1.45 (±0.04)	1.45 (±0.03)	0.76 (±5)	1.23 (±0.08)	0.67 (±0.03)
SNR (dB)	4.64 (±0.53)	4.53 (±0.38)	6.45 (±0.12)	6.07 (±0.07)	4.01 (±0.16)	4.88 (±0.5)	4.33 (±0.33)
